# Classification of MSH6 Variants of Uncertain Significance Using Functional Assays

**DOI:** 10.3390/ijms22168627

**Published:** 2021-08-11

**Authors:** Jane H. Frederiksen, Sara B. Jensen, Zeynep Tümer, Thomas v. O. Hansen

**Affiliations:** 1 Department of Clinical Genetics, Copenhagen University Hospital, Rigshospitalet, DK-2100 Copenhagen, Denmark; sara.bisgaard.jensen@regionh.dk (S.B.J.); asuman.zeynep.tuemer@regionh.dk (Z.T.); 2 Department of Pediatrics and Adolescent Medicine, Copenhagen University Hospital, Rigshospitalet, DK-2100 Copenhagen, Denmark; 3 Department of Clinical Medicine, Faculty of Health and Medical Sciences, University of Copenhagen, DK-2100 Copenhagen, Denmark

**Keywords:** functional assay, Lynch syndrome, MSH6, mismatch repair, variant of uncertain significance, variant interpretation, variant classification

## Abstract

Lynch syndrome (LS) is one of the most common hereditary cancer predisposition syndromes worldwide. Individuals with LS have a high risk of developing colorectal or endometrial cancer, as well as several other cancers. LS is caused by autosomal dominant pathogenic variants in one of the DNA mismatch repair (MMR) genes *MLH1*, *MSH2*, *PMS2* or *MSH6*, and typically include truncating variants, such as frameshift, nonsense or splicing variants. However, a significant number of missense, intronic, or silent variants, or small in-frame insertions/deletions, are detected during genetic screening of the MMR genes. The clinical effects of these variants are often more difficult to predict, and a large fraction of these variants are classified as variants of uncertain significance (VUS). It is pivotal for the clinical management of LS patients to have a clear genetic diagnosis, since patients benefit widely from screening, preventive and personal therapeutic measures. Moreover, in families where a pathogenic variant is identified, testing can be offered to family members, where non-carriers can be spared frequent surveillance, while carriers can be included in cancer surveillance programs. It is therefore important to reclassify VUSs, and, in this regard, functional assays can provide insight into the effect of a variant on the protein or mRNA level. Here, we briefly describe the disorders that are related to MMR deficiency, as well as the structure and function of MSH6. Moreover, we review the functional assays that are used to examine VUS identified in MSH6 and discuss the results obtained in relation to the ACMG/AMP PS3/BS3 criterion. We also provide a compiled list of the MSH6 variants examined by these assays. Finally, we provide a future perspective on high-throughput functional analyses with specific emphasis on the MMR genes.

## 1. Disorders of MMR Deficiency

The integrity of the human genome is continuously challenged by exogenous and endogenous factors that cause DNA damage. Endogenous factors include errors committed by the replicative polymerases, which misincorporate nucleotides, leading to base-base mismatches, as well as insertion/deletion loops that occur when the polymerase slip at repeat sequences, such as microsatellites. The DNA mismatch repair (MMR) system corrects these mismatches, as well as the insertions/deletions [1]. The core proteins of the mammalian MMR mechanism are MLH1 (mutL homolog 1), MSH2 (mutS homolog 2), PMS2 (postmeiotic segregation increased homolog 2), and MSH6 (mutS homolog 6). If the MMR mechanism is compromised, e.g., due to pathogenic variants in the core genes, the mismatched nucleotides will persist as mutations in the next round of replication, resulting in a hypermutator phenotype, including microsatellite instability, increasing the risk of developing cancer in specific tissues [1,2,3].

Heterozygous pathogenic variants in the MMR genes predispose to Lynch syndrome (LS, OMIM #120435), also known as hereditary nonpolyposis colorectal cancer (HNPCC), while homozygous or compound heterozygous pathogenic variants lead to constitutional MMR deficiency (CMMRD, OMIM #276300) syndrome. While LS is one of the most common hereditary cancer predisposition syndromes, with a frequency of 1:300 individuals [4], CMMRD is very rare [5]. Individuals with a pathogenic MMR variant have a high risk of developing early onset cancer, especially colorectal cancer (CRC) and endometrial cancer [6,7]. However, the risk of developing ovarian, stomach, hepatobiliary tract, urologic tract, small bowel, pancreatic, breast, prostate, and adrenocortical cancers, as well as glioblastomas, is also increased [8,9,10,11,12,13,14,15]. The cancer spectrum of CMMRD is quite different from LS, and includes brain tumors, digestive tract cancer, hematological malignancies, besides the mentioned LS-associated cancers [16]. Individuals with CMMRD often present with café-au-lait macules, as well as multiple tumors during childhood and adolescence [16]. In LS, the most commonly mutated genes are *MLH1* and *MSH2*, whereas most variants in CMMRD are found in PMS2 and, to a lesser extent, in MSH6, followed by MLH1 and MSH2 [5]. The first pathogenic MSH6 variant was reported in 1997 in a family with multiple LS-spectrum tumors [17]. Since then, several studies have shown that individuals with pathogenic MSH6 variants have a lower risk and later onset of CRC compared to MLH1 and MSH2 variants. At age 70 years, the CRC risk for MSH6 carriers is 20% in males and 12% in females, whereas the risk of CRC for male and female MLH1 carriers is 44% and 53%, respectively, and for male and female MSH2 carriers it is 42% and 45%, respectively [18]. In contrast, the cumulative risk at 70 years for endometrial cancer is 41%, which is comparable to MLH1 and MSH2 female carriers [18].

## 2. MSH6 Structure

The *MSH6* gene is located on chromosome 2, spanning 24 kb genomic sequence and consists of 10 exons, encoding a 1360 amino acid (aa) protein. The MSH6 protein has the following five functional domains: the mismatch binding, connector, lever, clamp, and ATPase domains. MSH6 is pseudosymmetric with MSH2, which has a similar domain structure [19]. Even though the MMR pathway is highly conserved from prokaryotes to eukaryotes, including the five-domain architecture, the aa sequence of human MSH6 is only 24% identical to those of E. coli in conserved regions [19]. In addition, the N-terminal of the human MSH6 protein is extended by 400 aa compared to the E. coli protein. The function of this region has been of high interest, since it seems to have few signature motifs [19]. However, the region contains the proliferating cell nuclear antigen (PCNA)-binding motif, also termed the PIP box (21)QXX(L/I)XXFF(28), which is conserved across mammalian species [20,21]. The interaction of MSH6 with PCNA tethers the MMR machinery to the replication machinery [22]. Moreover, the N-terminal contains several nuclear localization signals (NLS) that are conserved in higher eukaryotes. The NLS comprise three monopartite signals, NLS1 (246)KKRR(249), NLS2 (298)RKRKR(302), and NLS3 (311)KRK(313), and one putative bipartite sequence that combines NLS2 and 3 (298)RKRKRMVTGNGSLKRK(313) [23].

MSH6 interacts with MSH2 at two regions, aa 326 to 575 and aa 953 to 1360, and form the MutSα heterodimer [24]. The heterodimer has a θ (Theta)-like structure containing two channels, with the DNA helix running through one. The mismatch binding domain, which includes aa 362 to 518 of MSH6, is located between the two channels. When the MutSα complex binds the mismatched DNA, a conserved Phe-X-Glu motif at aa 432 to 434 of MSH6 interacts directly with the mismatched DNA [19,25]. Glu434 hydrogen bonds to the mismatched (nascent) nucleotide, which is wedged between Phe432 and Met459. The nucleotide of the mismatch on the other (original) DNA strand hydrogen bonds with Val429. These bonds, as well as other nonspecific protein:DNA interactions, opens the minor groove of the DNA helix, resulting in the DNA being kinked [26,27]. Some mismatches, such as C:C, do not hydrogen bond to Glu434. However, it has been suggested that the nonspecific protein:DNA interactions are sufficient to bend the DNA [19]. The connector domain (aa 519 to 717) is located between the lever domain and the ATPase domain in the tertiary structure of the protein [19]. The location of the connector domain suggests involvement in allosteric signaling between the lever and ATPase domains. The sequence conservation of the lever domain (aa 718 to 934 and 1009 to 1075) is quite low, except for one loop (aa 757 to 782). This conserved loop lies in the intersection of the connector, lever and ATPase domains, as well as a strikingly long α-helix of the lever domain, which spans much of the MSH6 secondary structure. It has been suggested that this loop might be involved in signaling between the DNA-binding and ATPase domains [19]. Ile972, Arg974, Asn975, Thr999, Lys1000 and Lys1004 in the clamp domain (aa 935 to 1008) make extensive nonspecific interactions with the DNA near the mismatch, as mentioned above [19]. The ATPase domain (aa 1076 to 1355) of MSH6 is highly conserved compared to the rest of MSH6. Both MSH6 and MSH2 have an ATPase domain, where the adenosine binding site in one monomer consists of a Walker A and Walker B motif, as well as an ABC signature motif from the other monomer. Several studies have indicated a difference in nucleotide affinity between the ATPase domains of the two proteins [19,28]. Other studies have shown that the binding of ATP drives conformational changes in the mismatch binding domain, located between the two channels, which result in the two channels of MutSα being fused into a single larger channel [25].

## 3. MSH6 Function

**Figure 1:**
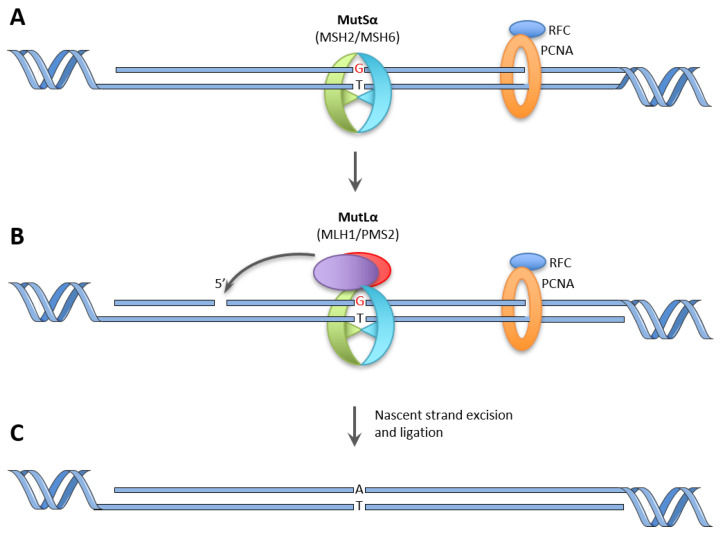
Overview of the mismatch repair pathway. (**A**) recognition of the mismatch by the MutSα. complex oriented by PCNA and RFC. (**B**) recruitment of the MutLα complex and nicking of the DNA 5’ to the mismatch by PMS2. (**C**) nascent strand excision and resynthesis, as well as final ligation of the nick.

The MutSα complex is responsible for mismatch recognition (Figure 1A). This complex mainly recognizes single-base mismatches and small insertion/deletion loops. MutSα and MutSβ (consisting of MSH2 and MSH3) are partly redundant, where MutSβ preferentially binds to larger insertion/deletion loops. In eukaryotic cells, the strand discrimination signal is a nick in the DNA, e.g., at the end of an Okazaki fragment. At this nick, PCNA is loaded onto the DNA strand by the protein replication factor C (RFC). PCNA is always oriented in the same direction on the DNA, and the orientation of PCNA will communicate the strand discrimination signal to the MMR machinery [29]. The details of communication between the strand discrimination signal and the MutSα complex are still not fully understood, and several models have been proposed [25,29,30]. After mismatch interaction, an exchange of ADP to ATP in MutSα causes several conformational changes, which, among others, cause MutSα to be able to interact with the MutLα complex (MLH1 and PMS2 heterodimer). A DNA nick 3’ of the mismatch causes activation of the endonuclease activity of PMS2, thereby creating a nick 5’ of the mismatch that is essential for the exonuclease EXO1 (Figure 1B) [29,31,32]. The newly synthesized strand including the mismatch is removed by EXO1, or through strand displacement by polymerase δ [33,34,35]. The EXO1-dependent pathway is the most dominant, where the emerging single-stranded DNA is stabilized by replication protein A (RPA), and polymerase δ resynthesizes the strand. Ligase I then seals the final nick, thereby completing the MMR (Figure 1C).

## 4. Variant Interpretation

Genetic testing has evolved from small gene panels based on phenotype to multigene panel testing, exome and whole-genome sequencing on a broader spectrum of cancer patients, which has resulted in an increase in the number of variants that need to be classified. Variants such as frameshifts and nonsense are relatively easy to interpret and are often classified as pathogenic. Unfortunately, a significant number of identified variants are missense, intron or silent variants, or small in-frame insertion/deletions, where the biological effects are more difficult to predict. Several tools can be used in the evaluation of MMR variants, including prediction programs that estimate the effect of a variant on the protein or mRNA level, databases with population frequencies, as well as databases such as the International Society for Gastrointestinal Hereditary Tumours (InSiGHT) and ClinVar (Table 1). InSiGHT curates a large database on genetic variants that are associated with gastrointestinal cancer, including MSH6 variants. The curators of the database are supported by a panel of experts in the field, who review the available data on a variant and recommend classification based on defined criteria. The classification of a variant by InSiGHT is therefore a very strong tool when interpreting the pathogenicity of a variant in the *MSH6* gene.

In 2008, a five-tier variant classification scheme was created, which classified benign variants as class 1, likely benign variants as class 2, variants of uncertain significance (VUS) as class 3, likely pathogenic variants as class 4, and pathogenic variants as class 5 [36]. The scheme was later adopted by InSiGHT [37], and with minor modifications by the American College of Medical Genetics and Genomics (ACMG)/Association for Molecular Pathology (AMP) [38]. This has led to a set of guidelines that are used for the interpretation of sequence variants [38]. These guidelines serve as a backbone for more gene-specific ACMG guidelines that are currently being generated for the MMR genes in the InSiGHT/ClinGen framework.

Unless substantial evidence points towards the variant being either pathogenic or non-pathogenic, the variant is—as stated above—classified as a VUS. Since the cancer risk of a VUS in the MMR genes is unknown, the clinical management of an individual with this type of variant is difficult, and relatives are not offered predictive testing. The *MSH6* gene has been reported to have a higher number of VUS compared to the other MMR genes [39], and currently 2935 MSH6 VUSs are reported in ClinVar (as of April 27th 2021). Therefore, there is an urgent need for reliable functional assays to gather more evidence to reclassify VUS in the *MSH6* gene.

In the following sections, we describe different functional assays that have been used for the reclassification of VUS identified in MSH6.

## 5. MMR Activity Assays

On the protein level, the key functional assay for variants in MSH6 is to measure the effect on MMR activity. Several, rather similar, in vitro MMR activity assays have been developed through the years, which all include a circular DNA substrate with a base:base mismatch or a small insertion in an endonuclease recognition site, as well as a single-stranded nick to direct the repair process. The MMR substrate is then added to variant MSH6 protein supplemented with wt MSH2, as well as cellular extract from an MSH6-deficient cell line. The correction of the mismatch will re-generate the cleavage site, and the degree of MMR can subsequently be determined by gel electrophoresis and compared to the MMR activity of wt MSH6. In total, we have collected data from nine MSH6 MMR activity publications from three different research groups.

**Figure 2:**
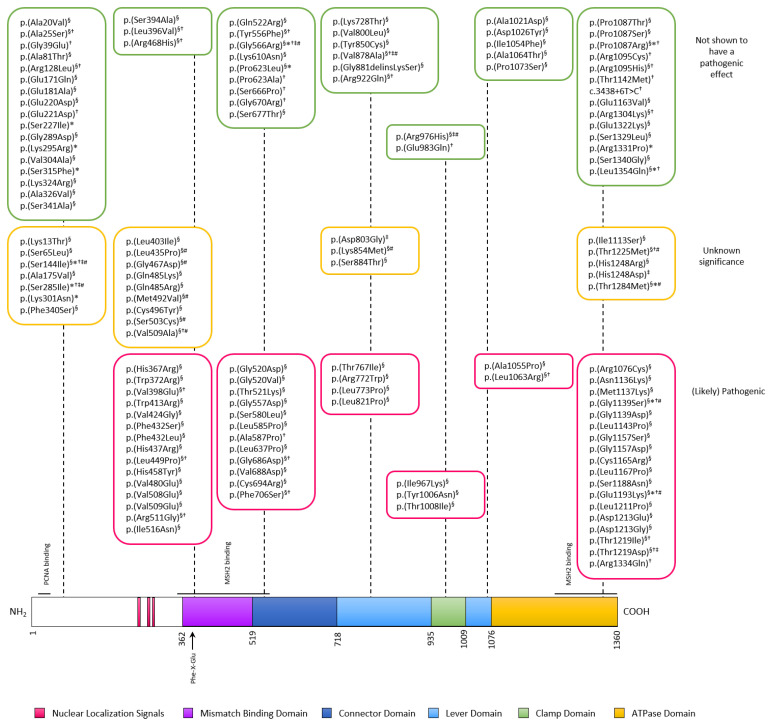
Structure of the MSH6 protein showing functional domains and the variants located within them on which functional analysis have been performed. The variants have been grouped according to the results of the functional assays. “Not shown to have a pathogenic effect”: variants that perform similarly to the wild type in the functional assays (green boxes). “Unknown significance”: variants that perform intermediate in the functional assays or give conflicting interpretations (yellow boxes). “(Likely) Pathogenic”: Variants with a functional effect (red boxes). The variants are marked to show which assays have been used: ^§^: MMR activity assays, *: nuclear localization assays, ^†^: 6-TG selection assays in mESC and ^‡^: mechanistic assays, ^#^: variants that give rise to conflicting interpretations in different assays.

The Nyström group used an MMR substrate that was developed from the pGEM phagemid, with a total extract of Sf9 insect cells overexpressing the MSH6 variant as well as wt MSH2 proteins, and supplemented this with nuclear extract from MSH6-deficient HCT15 or LoVo cells. As negative controls, nuclear extract from MMR-proficient TK6 or HeLa cells, as well as the MMR-deficient cell line supplemented with purified wt MSH6, were used. The group analyzed the MMR activity of 16 MSH6 variants (Figure 2 and Table S1). Four of the analyzed variants (p.(Leu435Pro, p.(Leu585Pro), p.(Val878Ala) and p.(Ser1188Asn)) showed decreased MMR activity [40,41,42,43,44].

In 2012, Geng et al. analyzed the activity of a single MSH6 variant, p.(Thr1219Asp), using an assay based on an MMR substrate that was derived from Wang and Hays [45,46], as well as nuclear extract from LoVo cells. The variant MSH6 and wt MSH2 proteins were purified from transduced High Five insect cells overexpressing the proteins. Wt MSH6 and nuclear extract from HeLa cells were used as negative controls. Geng et al. found the variant p.(Thr1219Asp) to be unable to perform MMR [47].

In 2012, Drost et al. published their first assay, in which they measured the activity of MSH6 variants [48]. Similarly to Geng et al., this assay also used an MMR substrate from Wang and Hays [45], which the group had further developed, as well as nuclear extract from LoVo cells. In contrast to Nyström and Geng, the variant proteins were produced by in vitro protein expression. For measurement and quantification of the MMR activity, Drost et al. used capillary electrophoresis, whereas Nyström and Geng used agarose gel electrophoresis [41,47]. As an evaluation of the assay, two repair-deficient variants, p.(Gly1139Ser) and p.(Thr1219Ile), were used, as well as p.(Ser144Ile), p.(Pro1087Arg), and p.(Pro1087Thr), which were shown by the Nyström group to be repair proficient [44]. By use of this assay the MMR activity of 20 MSH6 variants were measured and all were shown to be MMR proficient (Figure 2 and Table S1). Drost et al. continued to develop the MMR activity assay into the cell-free in vitro MMR activity (CIMRA) assay, which was first published for MLH1 and MSH2 variants in 2019 [49]. This assay has recently been further developed to include variants in MSH6 [50]. For this assay, Drost et al. created MSH2 and MSH6 double-deficient HeLa cells for nuclear extract. A two-component procedure was then developed to integrate the CIMRA data with computational predictions of pathogenicity, by using a set of 24 MSH6 variants that were classified by InSiGHT as either pathogenic or benign, as well as seven VUS. Drost et al. then continued to validate the two-component procedure using 18 MSH6 variants that were classified as (likely) benign by InSiGHT or ClinVar [50]. Unfortunately, Drost et al. had used all the pathogenic missense variants that were classified by InSiGHT and ClinVar for the calibration. To generate more pathogenic variants, a genetic screen of murine embryonic stem cells (mESC) was used in order to identify 38 inactivating variants where the aa are conserved between mice and humans. The results from the two-component analysis classified 35 of these variants as (likely) pathogenic, and three as VUS. Most importantly, none of the variants were falsely classified as (likely) benign. In total, Drost et al. analyzed 87 variants using the two-component procedure that were largely concordant with the expected outcome. The sensitivity and specificity of the two-component classification procedure showed a sensitivity of classification as (likely) pathogenic of 0.92, with a specificity of 1.00. For (likely) benign, the sensitivity was 0.78, with a specificity of 1.00. The reproducibility of the CIMRA assay was tested in three independent laboratories, which showed the assay to be highly reproducible [50]. The variant p.(Ser144Ile) had been previously determined to be MMR proficient by the Nyström group, and was used as a control by Drost in 2012 [44,48]. In accordance with these findings, the CIMRA assay showed the variant to be MMR proficient, and, interestingly, after applying the regression analysis to calculate the posterior probability, the variant was classified as VUS. Similarly, the variants p.(Ser503Cys), p.(Val509Ala), p.(Lys854Met), and p.(Thr1225Met) were found to have MMR activities close to wt MSH6 in both the Drost assay from 2012 and CIMRA. However, the posterior probability caused the variants to be classified as VUS. The variant p.(Ile1113Ser) had an MMR activity of 23.7% of wt MSH6, by the CIMRA assay, but integration of the data with the computational predictions caused the variant to be classified as VUS. Recently, Thompson et al. analyzed ten MSH6 variants using the CIMRA assay [51], of which nine variants have been previously analyzed by other activity assays [41,48,50]. In accordance with these data, they classified three variants (p.(Gly686Asp), p.(Arg772Trp) and p.(Gly1157Ser)) as likely pathogenic. Moreover, they classified the p.(Arg1076Cys) variant as likely pathogenic. The p.(Leu435Pro) variant was shown to be MMR deficient by the CIMRA assay, which is in accordance with data from the Nyström group [41]; however, the variant was classified as VUS by Thompson et al. Likewise, the p.(Met492Val) variant had similar activity measurements as the Drost et al. 2012 assay, indicating that the variant is MMR proficient, but were classified as VUS. Interestingly, unlike data from Drost et al., p.(Ser503Cys) was classified by Thompson et al. as likely benign.

## 6. 6-Thioguanine Selection Assay in Murine Embryonic Stem Cells (mESCs)

The 6-thioguanine (6-TG) selection assay in mESCs was first developed to characterize MSH2 variants [52], and was later adapted for variants in MSH6 [53,54]. A genetic screen was performed in *Msh6*+/− mESCs, where one *Msh6* allele was inactivated by insertion of a puromycin resistance gene [55]. The assay takes advantage of the fact that mice and human MSH6 aa sequences share more than 86% identity [54].

The assay consists of the following four steps: (1) Site-directed mutagenesis is performed with antisense-oriented short single-stranded locked nucleic acid-modified DNA oligonucleotides (LMOs), to introduce variants of interest into *Msh6*+/− mESCs. (2) The mESCs are then subjected to selection with 6-TG, a purine analog that, when incorporated, creates lesions that are recognized by the MMR system. However, the MMR machinery cannot repair the lesions, resulting in replication arrest and cell death in MMR-proficient cells, but not in MMR-deficient cells [39,56,57]. (3) To exclude loss of heterozygosity (LOH), a PCR analysis that detects both *Msh6* alleles was performed. (4) Finally, 6-TG-resistant mESCs that maintained both *Msh6* alleles were sequenced, to confirm the presence of the variant of interest. If no 6-TG-resistant mESC colonies contained the desired variant, the screen was repeated with a sense-oriented LMO.

The 6-TG selection assay was validated by examining three pathogenic, and four benign variants that were classified by the InSiGHT expert panel, as well as two variants that were classified by InSiGHT as VUSs (classified on 5 September 2013), but previously classified as pathogenic and non-pathogenic, respectively, using the 6-TG selection assay [53]. The assay detected all four pathogenic/putative pathogenic variants, but none of the five benign/putative benign variants, indicating that the assay can distinguish between pathogenic and benign variants.

Next, a total of 28 VUS, consisting of 27 missense variants and one intron variant (c.3438+6T>C), selected from the literature, the InSiGHT database or a clinical cohort, were investigated [53,54]. Of these, eight, (p.(Arg511Gly), p.(Ala587Pro), p.(Gly686Asp), p.(Phe706Ser), p.(Leu1063Arg), p.(Glu1193Lys), p.(Thr1219Asp), and p.(Thr1219Ile)), were identified in colonies with the desired sequence, and hence were classified as pathogenic. In line with these data, p.(Phe706Ser) and p.(Leu1063Arg) are now classified by InSiGHT as likely pathogenic and pathogenic, respectively, while p.(Gly686Asp), p.(Glu1193Lys), and p.(Thr1219Ile) are classified as likely pathogenic or pathogenic in ClinVar.

## 7. Nuclear Localization Assay

In agreement with its role in MMR, MSH6 is shown by immunohistochemistry analysis to be predominantly localized within the nucleus [58]. During S-phase, MSH6 is diffusely distributed throughout the nucleus; however, during an unperturbed S-phase, a population of MSH6 is localized to the nucleolus and this population is significantly reduced after DNA damage, suggesting that the protein is shuttled out of the nucleolus in response to damage [59]. The nuclear import of MSH6 is suggested to depend on dimerization with MSH2 in yeast [60,61]. However, studies using GFP-tagged MSH6 have suggested that this is not the case in human cells [23]. As mentioned above, MSH6 contains three nuclear localization sequences (NLSs) in the N-terminal region, which all seem to contribute to the subcellular localization of MSH6 [23].

To examine whether a variant influences the subcellular location of MSH6, the localization of a fluorescent protein-tagged variant and wild type (wt) MSH6 in transfected cells can be examined by confocal laser scanning microscopy. The variants are then scored as nuclear, cytosolic, or nuclear/cytosolic. This has been carried out for several MLH1 and MSH2 variants [62,63]. Currently, 14 MSH6 missense variants have been examined, using MSH6 tagged with GFP, either N- or C-terminally [23,64]. Twelve variants revealed localization similar to wt MSH6 (Figure 2 and Table S1). The p.(Ser285Ile) variant was shown to be distributed more equally between the nucleus and the cytoplasm in one study [23], while the variant behaved as wt MSH6 in another study [64]. None of the examined clinical variants are located in the putative NLSs. However, an artificial variant that is located in NLS2 p.(Lys301Asn), which later was reported in ClinVar as a VUS, showed increased cytoplasmic expression [64], suggesting that the NLS2 is important for nuclear localization of MSH6. Nevertheless, overall conformational changes may affect the cellular localization of MSH6, which suggests that localization studies of variants outside the NLS could be relevant.

## 8. Mechanistic Assays

Two papers, by Cyr and Heinen, and Geng et al., present an array of biochemical assays to evaluate the specific mechanistic malfunctions of MSH6 variants [47,65]. The techniques used by the two groups share a lot of similarities.

Geng et al. studied a single MSH6 variant, p.(Thr1219Asp), together with wt MSH2 purified from co-expressed Sf9 insect cells [47]. They found that the variant had no detectable MMR activity, as described above, and set out to investigate at which step the variant malfunctioned. Electrophoretic mobility shift assay (EMSA) and fluorescence anisotropy showed that the variant could bind the DNA mismatch, but, in contrast to wt, the binding was insensitive to ATP. To analyze the excision step of MMR (Figure 1), an assay resembling the MMR activity assay by Geng et al. was used. The results showed that the variant p.(Thr1219Asp) was completely deficient in excising the mismatched base, as it failed to support either 5′- or 3′-nick-directed excision. Geng et al. also found that the ATPase activity of p.(Thr1219Asp) was ten times higher than for wt MSH6, but dramatically lowered in the presence of a mismatched DNA substrate. Fluorescence anisotropy showed that MutSα containing MSH6 p.(Thr1219Asp) was less effective in exchanging ADP for ATP upon mismatch binding, and also had a lower ADP association and dissociation compared to wt. Using surface plasmon resonance (SPR), the ability to form a sliding clamp was examined through the association and dissociation to unblocked DNA. The results showed that MutSα p.(Thr1219Asp) was defective in the ability to form an ATP-dependent sliding clamp. SPR also showed that p.(Thr1219Asp) completely disrupts the formation of ternary DNA–MutSα–MutLα complexes, which is consistent with the inability to support 3’ nicked excision. Taken together, these data showed that MSH6 p.(Thr1219Asp) could bind mismatched DNA and hydrolyze ATP, but that these processes were not coupled as in the wt, and the procedures of ADP-to-ATP and sliding clamp formation and DNA–MutSα–MutLα complex formation were compromised, leading to a lack of MMR activity.

Cyr and Heinen investigated seven MSH6 missense variants ((p.(Ser144Ile), p.(Ser285Ile), p.(Gly566Arg), p.(Asp803Gly), p.(Val878Ala), p.(Arg976His), and p.(His1248Asp)) [65]. Similarly to Geng et al., the group purified the variant and wt MSH6 protein together with wt MSH2 from co-expressed insect cells. Cyr and Heinen analyzed the dimer-to-monomer ratio and found no effect of the variants on MutSα stability. The ATPase activity of the variants was then measured in the absence of DNA, where p.(Val878Ala) and p.(His1248Asp) seemed more severely affected. In the presence of mismatched DNA, p.(Gly566Arg), p.(Asp803Gly), p.(Val878Ala), p.(Arg976His), and p.(His1248Asp) had a more dramatic reduction in mismatch-stimulated ATPase. Of the seven variants, two, p.(Arg976His) and p.(His1248Asp), were by means of SPR found to have reduced affinity for mismatched DNA. Filter-binding experiments were used to investigate ADP and ATP binding. Three of the variant MutSα showed altered ADP binding compared to wt. Further, p.(Arg976His) and p.(His1248Asp) had reduced binding ability, whereas, interestingly, Cyr and Heinen found that the p.(Val878Ala) heterodimer could bind two molecules of ADP, in contrast to wt, which binds approximately one. With regards to ATP binding, p.(Ser285Ile), p.(Asp803Gly), p.(Arg976His), and p.(His1248Asp) were shown to be less efficient than wt, and p.(Gly566Arg) exhibited severely deficient ATP binding. By crosslinking experiments, it was found that the p.(Asp803Gly) also affects ATP binding negatively for the MSH2 subunit. When investigating the rate of the mismatch-stimulated exchange of ADP for ATP, p.(Arg976His) showed delayed release of ADP. By use of SPR, it was found that p.(Gly566Arg) and p.(Asp803Gly), despite their inability to properly bind ATP, are still able to dissociate from the mismatch DNA in the presence of high levels of ATP. The group then investigated the ATP-dependent conformational change by partial proteolytic digestion and mass spectrometry. The results indicated that p.(Gly566Arg) and p.(Asp803Gly) had an altered conformation, and more accessible structure of the ATPase and lever domains, and that MSH6 variants affect the conformation of both MSH6 and MSH2.

Overall, Cyr and Heinen found that five of the seven investigated MSH6 variants affect steps in the MMR mechanism (Table S1). However, the variants p.(Gly566Arg), p.(Val878Ala), p.(Arg976His), and p.(His1248Asp) were shown by other assays to be MMR proficient [44,48,50,54,64], and p.(Val878Ala) has also been reported as benign in ClinVar and as class 1 by the InSiGHT expert panel.

## 9. RNA Splicing Analysis

Based on in silico data, it is estimated that approximately 15%–25% of exonic, pathogenic variants mediate their effect via altered RNA splicing [66,67,68]. This is confirmed by functional studies of MLH1, which show that a high proportion of exon variants affect splicing [69,70]. RNA studies are therefore crucial in clinical assessment, and recent data suggest that RNA analysis can significantly reduce the number of variants that are classified as VUS [71].

The most common splicing aberration involves disruption of the canonical splice donor or splice acceptor sites. Such variants may result in exon skipping. Variants might also activate cryptic splice sites or create *de novo* splice sites, resulting in partial exon skipping or partial exon inclusion, while other variants result in intron retention [72,73].

**Figure 3:**
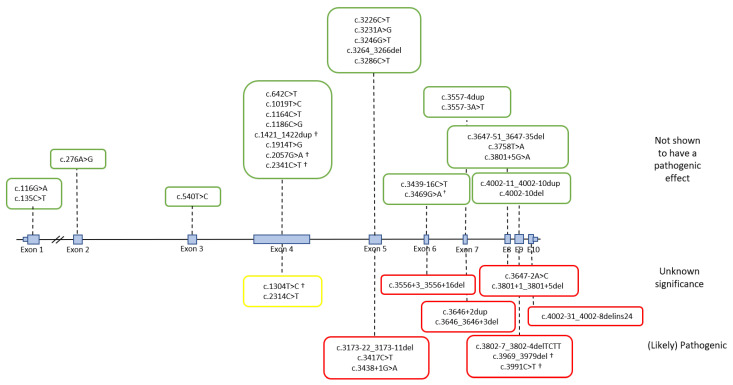
Structure of the *MSH6* gene showing the 10 exons and the variants located within or close to them on which published splicing analysis have been performed. The variants have been grouped according to the results of the splicing assays. No effect on splicing (green boxes); incomplete splicing (yellow box); splicing effect (red boxes). Variants marked with † indicates truncating variants or variants shown to have an effect on the protein level.

In silico splicing prediction tools are widely used to predict the effect of a variant on splicing (Table 1), and are helpful in variant prioritization for RNA analysis. However, most splicing tools are designed to predict alteration at donor and acceptor splice sites, and their predictive values are lower for variants in other areas. Therefore, functional analysis is needed in order to establish whether a variant is associated with aberrant splicing. In contrast to MLH1 and MSH2, only a small fraction of MSH6 variants have been studied with RNA analyses, and few variants have been reported to affect splicing (Table S1). MSH6 splicing analyses have been performed by examining RNA in whole blood or other tissues from variant carriers, by reverse transcription PCR (RT-PCR) and long-range RT-PCR analysis [37,51,71,74,75,76,77,78,79,80,81,82,83,84], and, more recently, by targeted RNA sequencing [71,75]. Moreover, in cases where RNA is not available, minigene splicing assays have been performed [76,77]. The 40 MSH6 variants that were collected in this review are published data, and include 16 intronic variants, 11 missense variants, nine synonymous variants, two frameshift variants, one nonsense variant, and one 3-bp in-frame deletion located in various exons and introns of MSH6 (Figure 3). RNA analyses showed that seven intronic variants (c.3173-22_3173-11del, c.3438+1G>A, c.3556+3_3556+16del, c.3646_3646+3del, c.3646+2dup, c.3801+1_3801+5del, and c.3802-7_3802-4delTCTT) induced exon skipping, resulting in either a frameshift and a premature stop codon, or an in-frame deletion (exon 7) in a functional domain (ATPase domain), while two intron variants induced intron retention (c.3647-2A>C and c.4002-31_4002-8delins24). Two missense variants (c.1304T>C, p.(Leu435Pro) and c.2314C>T, p.Arg772Trp)), both located in exon 4, were reported to induce skipping; however, the results should be regarded as incomplete, since skipping of exon 4 is also observed in control samples [51]. This points to the fact that when interpreting RNA results, it is important to know the contribution of naturally occurring isoforms. Recent studies have identified several different *MSH6* transcript isoforms [74,75,85,86], including isoforms with cassette events, such as the skipping of exons 2 (resulting in frameshift), exon 3 (frameshift), or exon 4 (frameshift); multicasette events, such as the skipping of exons 2 and 3 (frameshift), or skipping of exons 3 and 4 (in-frame); or transcript isoforms using cryptic splice sites in various exons. The skipping of exon 4 is the most common naturally occurring transcript and is observed in up to 25% of the reference transcript expression.

Finally, one synonymous variant (c.3417C>T, p.(Gly1139=)) resulted in the creation of a de novo splice donor site, while two truncating variants (c.3969_3979del, p.(Phe1323Leufs*14) and c.3991C>T, p.(Arg1331*)) interestingly resulted in exon skipping, by affecting yet undefined exonic splicing enhancer elements (Table S1).

## 10. Discussion

MSH6 germline variants can be classified by clinical data, such as co-segregation and tumor information (MSH2/MSH6 expression and microsatellite status), together with variant information. However, in many cases, this information is lacking, and additional information is needed before a variant can be classified as benign or pathogenic. Functional assays are therefore used to gather more information regarding specific variants.

In this review, we have collected 207 unique MSH6 variants that have been previously examined by functional analysis, including MMR activity, 6-TG selection, nuclear localization, mechanistic and RNA splicing assays. The pathogenic missense variants that are described are scattered throughout the different domains of the MSH6 protein. Most are located in the mismatch binding, the connector, and the ATPase domains (Figure 2). Strikingly, no pathogenic missense variants are currently reported in the N-terminal part of the MSH6 protein, questioning the importance of this domain, although variants of the few conserved amino acids might still be clinically relevant.

For all the assays, it is applicable that there are several points that need to be considered before data are included in the assessment of the pathogenicity of germline variants, including experimental design, replication, controls, and validation. Recommendations for the application of the functional evidence criteria PS3/BS3 using the ACMG/AMP guidelines has recently been published to clarify the variant interpretation process for functional assays [87].

Regarding the experimental design, all the assays model the pathogenesis mechanism. The MMR activity, nuclear localization and splicing assays, as well as the various mechanistic assays, are limited to the examination of one specific MSH6 protein function. This means that a test result that shows an effect on MSH6 function can be used in variant classification, whereas a negative test result cannot automatically be interpreted as benign. In contrast, the 6-TG selection assay is a survival assay, which has the advantage that the output includes all the biological functions of MSH6, as well as effects at the mRNA level. However, the 6-TG selection assay is performed in mouse ESCs, and careful consideration should be given when analyzing the functional effects of human variants in non-human cells.

The MMR activity (CIMRA) assay includes internal controls (wt and null variants), and is replicated and calibrated/validated using several known pathogenic and benign variants (≥11 in total) to determine the sensitivity and specificity of the assay (Table 2). Moreover, this assay also integrates the functional data with computational predictions of pathogenicity (OddsPath), and is therefore the only assay were the PS3_very_strong and BS3 criteria are fulfilled. The 6-TG selection assay described in Houlleberghs et al., 2017 also uses basic internal controls, but uses ≤10 benign/pathogenic variant controls, and currently the sensitivity and specificity of the assay are unknown. For this reason, the maximal criteria that apply for this assay are PS3 supporting/BS3 supporting. The same goes for the other MMR activity assays. In contrast, the nuclear localization and mechanistic assays described in this review do not include known null variants or known pathogenic/benign validation controls, and therefore the PS3/BS3 criteria is not met for these assays. In accordance, some discrepancy is observed when comparing results from the mechanistic assays with other assays and/or with clinical data.

The development of the nuclear localization assay as a quantitative functional assay is complex, since the amount of protein overexpression can affect protein mislocalization and misfolding. Moreover, before localization analysis using fusion proteins, it is important to examine the effect of tagged MSH6 proteins at C-terminal or N-terminal ends, since this may affect protein expression levels, cellular localization, and MMR activity, as previously shown for MLH1 and PMS2 [88].

Several MSH6 variants were examined by CIMRA and 6-TG selection functional assays with similar classification. However, currently there is no consensus on whether results from different functional assays could be combined. Therefore, if multiple functional assay results are available for a single variant, evidence from the assay that is the most validated and best measures the disease mechanism should be applied [87]. 

Regarding splicing assays, these are more difficult to validate, at least when RNA from patient samples is used. However, recommendations for RT-PCR protocols that are used in the clinical testing have previously been developed for BRCA1/BRCA2 variants [89], and these guidelines are readily adaptable to MMR variants. Interpretation of splicing results from intron and synonymous variants is more straightforward than missense and in-frame variants that may also have an effect on the protein level. Moreover, some variants introduce nonsense-mediated decay (NMD), while others are expected to shorten (in-frame deletion due to exon skipping) or truncate the protein. Therefore, NMD inhibitors are widely used during lymphoblast cell line culturing. Importantly, it is crucial to determine the proportion of alternative transcripts arising from a variant allele, and this is required by InSiGHT in order to classify variants using data from splicing assays. In agreement with results from BRCA1 studies, showing that a splice variant resulting in 70–80% expression of a non-functional allele does not result in an increased breast cancer risk [90], data from MSH2 suggest that >70% of non-functional transcript isoforms should be present before the variant can be classified as pathogenic [51,74].

## 11. Future Directions

The rapid increase in the number of VUS discovered due to novel sequencing techniques calls for development of large-scale functional assays. During the latest years, several studies of cancer pre-disposing genes, including BRCA1, PTEN, and TP53, using saturation mutagenesis, together with a high-throughput functional analysis, have been performed [91,92,93,94,95,96]. Most of these assays are specific for the gene function/pathway of interest. However, Matreyek et al. developed an assay called variant abundance by massively parallel sequencing (VAMP-seq), which can measure the effects of thousands of missense variants of a protein on intracellular abundance, simultaneously [96]. The assay is based on the estimation that up to 75% of the pathogenic variation in monogenic disease is due to disruption of thermodynamic stability, and, consequently, protein abundance [96,97,98]. The study applied their analysis to PTEN and TPMT, but their data suggest that the assay can also be used for MMR genes such as MLH1 and PMS2, although the stabilizing effects of the variants must be interpreted with caution, as previously suggested [99].

More recently, MSH2 was examined in a human cell line model, in which deletion of MSH2 was complemented with libraries of variants comprising nearly every possible MSH2 missense allele, covering 94% of the 17,746 possible variants [39]. The cells were then treated with 6-TG to select for MMR dysfunction (as described above), followed by deep sequencing to identify surviving MSH2 variants. The results were highly concordant (96%) with existing functional data and expert clinicians’ interpretations, and showed that the large majority (89%) of missense variants were functionally neutral. This assay can be directly implemented for the other MMR genes as well, including MSH6.

Recently, the atlas of variant effects (AVE) alliance, an international collaborative initiative, whose goal is to resolves issues relating to the use of multiplex assays of variant effect (MAVE) data to interpret human genetic variants (https://www.varianteffect.org, accessed on 25 May 2021), has been launched, and we anticipate that this effort may increase information regarding interpretation of variants considerably. If properly validated, we expect that data from these high-throughput assays will be incorporated in variant classification guidelines and clinical decision-making in the near future. The MSH6 variants that were examined by functional analysis, collected in this review, may provide a set of variants for validation of these future large-scale assays.

In relation to splicing, we expect that long-read RNA sequencing will be implemented in the genetic diagnostic pipeline within the next few years, enabling delineation of complex structures resulting from aberrant splicing, as well as co-occurrence of splicing events and allele-specific expression analysis, and providing a more comprehensive RNA splicing analysis.

## Figures and Tables

**Table 1:** Software and additional tools for evaluation of MSH6 variants.

Software	Description	Website
**CADD**	CADD is a freely available tool from the University of Washington and HudsonAlpha Institute for Biotechnology for scoring the deleteriousness of single-nucleotide variants as well as insertion/deletions variants in the human genome.	https://cadd.gs.washington.edu/snv (accessed on 24 May 2021)
**REVEL**	REVEL is a new ensemble method for predicting the pathogenicity of missense variants based on a combination of scores from the following 13 individual tools: MutPred, FATHMM v2.3, VEST 3.0, Polyphen-2, SIFT, PROVEAN, MutationAssessor, MutationTaster, LRT, GERP++, SiPhy, phyloP, and phastCons.	https://sites.google.com/site/revelgenomics/ (accessed on 24 May 2021)
**Polyphen2**	PolyPhen-2 (Polymorphism Phenotyping v2) is a tool from Harvard University which predicts possible impact of an amino acid substitution on the structure and function of a human protein.	http://genetics.bwh.harvard.edu/pph2/ (accessed on 24 May 2021)
**Align-GVGD**	Align-GVGD is a freely available, web-based program that combines the biophysical characteristics of amino acids and protein multiple sequence alignments to predict where missense substitutions in genes of interest fall in a spectrum from enriched deleterious to enriched neutral.	http://agvgd.hci.utah.edu/agvgd_input.php (accessed on 24 May 2021)
**HCI database of prior probability**	The Huntsman Cancer Institute Database of Prior Probabilities of Pathogenicity for single-nucleotide substitutions in cancer genes provides the prior probability of pathogenicity estimates for MSH6 variants.	http://priors.hci.utah.edu/PRIORS/ (accessed on 24 May 2021)
**SpliceAI**	SpliceAI is a deep learning-based tool from Broad Institute to identify splice variants.	https://spliceailookup.broadinstitute.org/ (accessed on 24 May 2021)
**MaxEntScan**	MaxEntScan is based on the approach for modeling the sequences of short sequence motifs such as those involved in RNA splicing, which simultaneously accounts for non-adjacent as well as adjacent dependencies between positions.	http://hollywood.mit.edu/burgelab/maxent/Xmaxentscan_scoreseq.html (accessed on 24 May 2021)
**gnomAD**	The Genome Aggregation Database (gnomAD) is a resource developed by an international coalition of investigators, with the goal of aggregating and harmonizing both exome and genome sequencing data from a wide variety of large-scale sequencing projects, and making summary data available for the wider scientific community.	https://gnomad.broadinstitute.org/ (accessed on 24 May 2021)
**Entrez-pubmed**	Web-based search engine to search for research publications containing specific variants.	http://www.ncbi.nlm.nih.gov/pubmed (accessed on 24 May 2021)
**InSiGHT variants databases**	InSiGHT houses and curates the most comprehensive database of DNA variants re-sequenced in the genes that contribute to gastrointestinal cancer.	https://www.insight-group.org/variants/databases/ (accessed on 24 May 2021)
**ClinVar**	ClinVar aggregates information about genomic variation and its relationship to human health.	https://www.ncbi.nlm.nih.gov/clinvar (accessed on 24 May 2021)

**Table 2:** Evaluation of functional studies examining MSH6 variants.

Assay	MMR Activity									6-TG Selection		Nuclear Localization		Mechanistic		Splicing
**Reference**	[44]	[43]	[42]	[40]	[48]	[41]	[47]	[50]	[51]	[53]	[54]	[23]	[64]	[65]	[47]	[51,71,74,75,76,77,78,79,80,81,82,83,84,85,86]
**Material used**	Human cDNA in baculovirus vector	Human cDNA in baculovirus vector	Human cDNA in baculovirus vector	Human cDNA in baculovirus vector	Human cDNA in pCITE4a	Human cDNA in baculovirus vector	Human cDNA in baculovirus vector	Human cDNA in pCITE4a	Human cDNA in pCITE4a	Mouse DNA	Mouse DNA	Human cDNA in EGFP-C1	Human cDNA in pEGFP-N1	Human cDNA in baculovirus vector	Human cDNA in baculovirus vector	Human mRNA or minigene DNA constructs
**Expression system**	Sf9 insect cells	Sf9 insect cells	Sf9 insect cells	Sf9 insect cells	IVTT	Sf9 insect cells	Sf9 insect cells	IVTT	IVTT	mESC	mESC	DLD-1 cells	HEK293 cells	Sf9 insect cells	Sf9 insect cells	RT-PCR
**Functional output**	Mismatch repair activity	Mismatch repair activity	Mismatch repair activity	Mismatch repair activity	Mismatch repair activity	Mismatch repair activity	Mismatch repair activity	Mismatch repair activity	Mismatch repair activity	Sequencing of 6TG resistant colonies	Sequencing of 6TG resistant colonies	Nuclear/cytoplasmic localization	Nuclear/cytoplasmic localization	Multiple outputs	Multiple outputs	cDNA sequence
**Wild-type control**	Yes	Yes	Yes	Yes	Yes	Yes	Yes	Yes	Yes	-	yes	yes	Yes	Yes	Yes	-
**Positive control**	Yes (HCT- 15 cells)	Yes (HCT- 15 cells)	Yes (HCT- 15 cells)	Yes (LoVo cells)	2 pathogenic variants	Yes (LoVo cells)	Yes (LoVo cells)	52 pathogenic variants	1 pathogenic variant	1 pathogenic variant	5 pathogenic variants	-	-	-	-	-
**Negative control**	Yes (TK6 cells)	Yes (TK6 cells)	Yes (TK6 cells)	Yes (HeLa cells)	3 benign variants	Yes (HeLa cells)	Yes (HeLa cells)	31 benign variants	-	-	4 benign variants	-	-	-	-	-
**No. of VUS examined**	4	2	5	1	15	6	1	7	10	3	26	4	10	7	1	-
**Technical replicates**	2	2	2	3	3-4 for VUS and >6 for controls	3	-	>3	3-4	-	-	-	3	3	3	-
**Biological replicates**	-	-	-	-	-	-	-	-	-	yes	yes	-	-	-	-	-
**PS3/BS3 criterion** [87]	Max PS3_Supporting/Max BS3_Supporting	Max PS3_Supporting/Max BS3_Supporting	Max PS3_Supporting/Max BS3_Supporting	Max PS3_Supporting/Max BS3_Supporting	Max PS3_Supporting/Max BS3_Supporting	Max PS3_Supporting/Max BS3_Supporting	Max PS3_Supporting/Max BS3_Supporting	Max PS3_Very_Strong/Max BS3	Max PS3_Very_Strong/Max BS3	Do no use PS3/BS3	Max PS3_Supporting/Max BS3_Supporting	Do no use PS3/BS3	Do no use PS3/BS3	Do no use PS3/BS3	Do no use PS3/BS3	Max PS3_Very_Strong/Max BS3

## Data Availability

All data came for published articles.

## Supplementary Materials

The following are available online at https://www.mdpi.com/article/10.3390/ijms22168627/s1.
